# 5-Amino-7-(4-bromo­phen­yl)indane-4,6-dicarbonitrile

**DOI:** 10.1107/S1600536809043426

**Published:** 2009-10-28

**Authors:** Cunlan Zhang

**Affiliations:** a Department of Chemistry, Dezhou University, Dezhou 253023, People’s Republic of China

## Abstract

In the title mol­ecule, C_17_H_12_BrN_3_, the mean planes of the bicyclic system and the attached aromatic ring form a dihedral angle of 63.12 (7)°. In the crystal structure, weak inter­molecular N—H⋯N hydrogen bonds link adjacent mol­ecules into ribbons extending along [010].

## Related literature

Analogous compounds have been synthesized and reported by Hafidh *et al.* (2002 ▶) and Hafidh & Zantour (2003 ▶). For a related structure, see Mereiter *et al.* (2000 ▶).
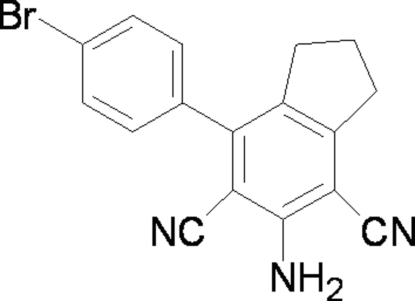


## Experimental

### 

#### Crystal data

C_17_H_12_BrN_3_
*M* *_r_* = 338.21
Orthorhombic, 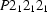

*a* = 7.5655 (14) Å
*b* = 11.811 (2) Å
*c* = 16.490 (3) Å
*V* = 1473.5 (5) Å^3^
*Z* = 4
Mo *K*α radiation
μ = 2.79 mm^−1^
*T* = 298 K
0.35 × 0.28 × 0.20 mm

#### Data collection

Bruker SMART 1000 CCD area-detector diffractometer
Absorption correction: multi-scan (*SADABS*; Sheldrick, 1996 ▶) *T*_min_ = 0.442, *T*_max_ = 0.606
7199 measured reflections
2708 independent reflections
2171 reflections with *I* > 2σ(*I*)
*R*_int_ = 0.027

#### Refinement

*R*[*F* ^2^ > 2σ(*F* ^2^)] = 0.030
*wR*(*F* ^2^) = 0.063
*S* = 0.95
2708 reflections
198 parameters
H atoms treated by a mixture of independent and constrained refinement
Δρ_max_ = 0.41 e Å^−3^
Δρ_min_ = −0.24 e Å^−3^
Absolute structure: Flack (1983 ▶), 1253 Friedel pairs
Flack parameter: 0.008 (10)

### 

Data collection: *SMART* (Siemens, 1996 ▶); cell refinement: *SAINT* (Siemens, 1996 ▶); data reduction: *SAINT*; program(s) used to solve structure: *SHELXS97* (Sheldrick, 2008 ▶); program(s) used to refine structure: *SHELXL97* (Sheldrick, 2008 ▶); molecular graphics: *SHELXTL* (Sheldrick, 2008 ▶); software used to prepare material for publication: *SHELXTL*.

## Supplementary Material

Crystal structure: contains datablocks I, global. DOI: 10.1107/S1600536809043426/cv2633sup1.cif

Structure factors: contains datablocks I. DOI: 10.1107/S1600536809043426/cv2633Isup2.hkl

Additional supplementary materials:  crystallographic information; 3D view; checkCIF report

## Figures and Tables

**Table 1:** Hydrogen-bond geometry (Å, °)

*D*—H⋯*A*	*D*—H	H⋯*A*	*D*⋯*A*	*D*—H⋯*A*
N1—H1*B*⋯N2^i^	0.85 (3)	2.40 (3)	3.227 (4)	165 (3)
N1—H1*A*⋯N3^ii^	0.82 (3)	2.46 (3)	3.247 (4)	161 (2)

Symmetry codes: (i) 
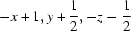
; (ii) 
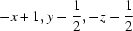
.

**Fig. 1.**
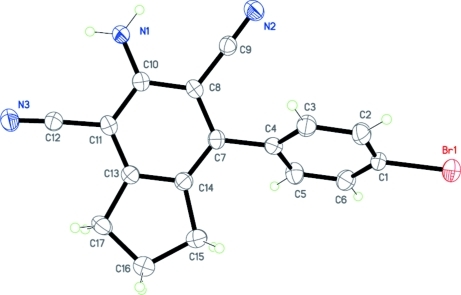
The molecular structure of (I). Displacement ellipsoids are drawn at the 30% probability level.

## supplementary crystallographic information

### Comment

indanee derivatives have attracted some attention in a search of novel functional compounds (Hafidh *et al.* 2002; Hafidh & Zantour, 2003). As our contribution to this field, we present here the title compound, (I).

In (I) (Fig. 1), all bond lengths and angles are normal and correspond to those observed in related compound (Mereiter *et al.*, 2000). The mean plane of the bicycle system (C7/C8/C10/C11/C13-C17) and attached aromatic ring (C1-C6) form a dihedral angle of 63.12 (7)°.

In the crystal structure, weak intermolecular N—H···N hydrogen bonds (Table 1) link adjacent molecules into ribbons extended in direction [010].

### Experimental

The malononitrile (1.32 g,20 mmol) was added into the mixture of 4-bromobenzaldehyde (1.85 g,10 mmol) and cyclopentanone (0.84 g,10 mmol) in 1-butyl-3-methylimidazol-3-ium tetrafluoroborate (20 ml), and has stirred for three hours at 388 K. The solution was allowed to stand for 2 weeks, whereupon the crystals suitable for the *X*-ray study was obtained. Yield: 1.047 g, 31%. Anal. for C_17_H_12_BrN_3_: Calc. C, 60.37; H, 3.58; N, 12.42; Found: C, 60.54; H, 3.71; N, 12.54%. The No. of CCDC: 750008.

### Refinement

C-bound H atoms were placed in geometrically idealized positions (C—H 0.93-0.97 Å) and treated as riding on their parent atoms , with *U*_iso_(H)=1.2*U*_eq_(C). Amino H atoms were located on a difference map and refined isotropically with the bond restraint of N—H = 0.84 (3) Å.

### Crystal data

**Table:**

C_17_H_12_BrN_3_	*D*_x_ = 1.525 Mg m^−^^3^
*M**_r_* = 338.21	Mo *K*α radiation, λ = 0.71073 Å
Orthorhombic, *P*2_1_2_1_2_1_	Cell parameters from 2622 reflections
*a* = 7.5655 (14) Å	θ = 2.5–23.4°
*b* = 11.811 (2) Å	µ = 2.79 mm^−^^1^
*c* = 16.490 (3) Å	*T* = 298 K
*V* = 1473.5 (5) Å^3^	Block, colourless
*Z* = 4	0.35 × 0.28 × 0.20 mm
*F*(000) = 680	

### Data collection

**Table:**

Bruker SMART 1000 CCD area-detector diffractometer	2708 independent reflections
Radiation source: fine-focus sealed tube	2171 reflections with *I* > 2σ(*I*)
graphite	*R*_int_ = 0.027
φ and ω scans	θ_max_ = 25.5°, θ_min_ = 2.1°
Absorption correction: multi-scan (*SADABS*; Sheldrick, 1996)	*h* = −9→8
*T*_min_ = 0.442, *T*_max_ = 0.606	*k* = −14→9
7199 measured reflections	*l* = −15→19

### Refinement

**Table:**

Refinement on *F*^2^	Secondary atom site location: difference Fourier map
Least-squares matrix: full	Hydrogen site location: inferred from neighbouring sites
*R*[*F*^2^ > 2σ(*F*^2^)] = 0.030	H atoms treated by a mixture of independent and constrained refinement
*wR*(*F*^2^) = 0.063	*w* = 1/[σ^2^(*F*_o_^2^) + (0.0269*P*)^2^] where *P* = (*F*_o_^2^ + 2*F*_c_^2^)/3
*S* = 0.95	(Δ/σ)_max_ < 0.001
2708 reflections	Δρ_max_ = 0.41 e Å^−^^3^
198 parameters	Δρ_min_ = −0.24 e Å^−^^3^
0 restraints	Absolute structure: Flack (1983), 1253 Friedel pairs
Primary atom site location: structure-invariant direct methods	Flack parameter: 0.008 (10)

### Special details

**Table:**

Geometry. All e.s.d.'s (except the e.s.d. in the dihedral angle between two l.s. planes) are estimated using the full covariance matrix. The cell e.s.d.'s are taken into account individually in the estimation of e.s.d.'s in distances, angles and torsion angles; correlations between e.s.d.'s in cell parameters are only used when they are defined by crystal symmetry. An approximate (isotropic) treatment of cell e.s.d.'s is used for estimating e.s.d.'s involving l.s. planes.
Refinement. Refinement of *F*^2^ against ALL reflections. The weighted *R*-factor *wR* and goodness of fit *S* are based on *F*^2^, conventional *R*-factors *R* are based on *F*, with *F* set to zero for negative *F*^2^. The threshold expression of *F*^2^ > σ(*F*^2^) is used only for calculating *R*-factors(gt) *etc*. and is not relevant to the choice of reflections for refinement. *R*-factors based on *F*^2^ are statistically about twice as large as those based on *F*, and *R*- factors based on ALL data will be even larger.

### Fractional atomic coordinates and isotropic or equivalent isotropic displacement parameters (Å^2^)

**Table:**

	*x*	*y*	*z*	*U*_iso_*/*U*_eq_	
Br1	0.61120 (5)	0.10058 (3)	0.17284 (2)	0.06606 (14)	
C1	0.6025 (5)	0.2417 (2)	0.11713 (14)	0.0449 (7)	
C2	0.7401 (4)	0.2717 (3)	0.06832 (18)	0.0530 (8)	
H2	0.8383	0.2249	0.0633	0.064*	
C3	0.7311 (4)	0.3727 (3)	0.02640 (18)	0.0484 (8)	
H3	0.8243	0.3937	−0.0072	0.058*	
C4	0.5870 (4)	0.4429 (2)	0.03340 (15)	0.0396 (7)	
C5	0.4503 (4)	0.4104 (3)	0.08498 (17)	0.0501 (7)	
H5	0.3533	0.4579	0.0918	0.060*	
C6	0.4572 (4)	0.3083 (3)	0.12627 (18)	0.0521 (8)	
H6	0.3643	0.2857	0.1595	0.062*	
C7	0.5761 (4)	0.5533 (2)	−0.00930 (15)	0.0374 (6)	
C8	0.5789 (4)	0.5572 (2)	−0.09608 (16)	0.0390 (7)	
C9	0.5889 (4)	0.4534 (2)	−0.14014 (16)	0.0418 (7)	
C10	0.5655 (4)	0.6602 (2)	−0.13850 (16)	0.0398 (7)	
C11	0.5607 (4)	0.7613 (2)	−0.09254 (16)	0.0405 (7)	
C12	0.5519 (4)	0.8699 (3)	−0.13046 (17)	0.0449 (8)	
C13	0.5621 (4)	0.7567 (2)	−0.00771 (16)	0.0403 (7)	
C14	0.5666 (3)	0.6538 (2)	0.03272 (15)	0.0399 (7)	
C15	0.5628 (4)	0.6735 (3)	0.12311 (16)	0.0524 (8)	
H15A	0.6629	0.6373	0.1492	0.063*	
H15B	0.4546	0.6441	0.1466	0.063*	
C16	0.5724 (5)	0.8015 (3)	0.13270 (18)	0.0554 (9)	
H16A	0.4762	0.8278	0.1667	0.066*	
H16B	0.6832	0.8228	0.1580	0.066*	
C17	0.5589 (4)	0.8542 (3)	0.04933 (17)	0.0507 (8)	
H17A	0.4498	0.8966	0.0438	0.061*	
H17B	0.6578	0.9045	0.0393	0.061*	
H1A	0.557 (3)	0.605 (3)	−0.2469 (16)	0.042 (8)*	
H1B	0.538 (4)	0.724 (3)	−0.2445 (18)	0.052 (10)*	
N1	0.5612 (4)	0.6630 (3)	−0.22009 (15)	0.0564 (8)	
N2	0.5985 (4)	0.3714 (2)	−0.17670 (16)	0.0587 (7)	
N3	0.5450 (4)	0.9583 (2)	−0.15788 (15)	0.0635 (8)	

### Atomic displacement parameters (Å^2^)

**Table:**

	*U*^11^	*U*^22^	*U*^33^	*U*^12^	*U*^13^	*U*^23^
Br1	0.1015 (3)	0.04187 (17)	0.05480 (18)	0.0033 (2)	0.0026 (2)	0.01060 (16)
C1	0.067 (2)	0.0317 (15)	0.0355 (14)	0.0015 (18)	−0.0058 (16)	0.0039 (11)
C2	0.060 (2)	0.046 (2)	0.0525 (19)	0.0155 (17)	0.0067 (16)	0.0055 (16)
C3	0.0497 (19)	0.046 (2)	0.0489 (18)	0.0075 (15)	0.0097 (14)	0.0064 (15)
C4	0.0448 (18)	0.0371 (15)	0.0368 (14)	−0.0012 (15)	0.0014 (14)	−0.0036 (12)
C5	0.0471 (18)	0.0439 (18)	0.0593 (18)	0.0037 (15)	0.0043 (14)	0.0052 (16)
C6	0.058 (2)	0.049 (2)	0.0496 (18)	−0.0066 (17)	0.0089 (15)	0.0064 (15)
C7	0.0343 (16)	0.0352 (15)	0.0428 (15)	−0.0003 (13)	−0.0004 (12)	0.0031 (12)
C8	0.0408 (17)	0.0320 (14)	0.0443 (15)	0.0018 (13)	−0.0001 (13)	−0.0031 (12)
C9	0.0455 (18)	0.0389 (17)	0.0412 (15)	0.0004 (17)	−0.0042 (14)	0.0032 (14)
C10	0.0401 (18)	0.0361 (16)	0.0431 (15)	−0.0013 (14)	−0.0004 (13)	0.0014 (13)
C11	0.0409 (18)	0.0325 (15)	0.0481 (16)	−0.0012 (13)	−0.0022 (13)	−0.0009 (13)
C12	0.055 (2)	0.0398 (18)	0.0402 (16)	−0.0030 (14)	−0.0039 (14)	0.0005 (14)
C13	0.0423 (18)	0.0380 (16)	0.0405 (15)	0.0023 (14)	0.0009 (13)	−0.0014 (13)
C14	0.0408 (17)	0.0400 (16)	0.0389 (15)	0.0048 (13)	0.0018 (13)	0.0001 (13)
C15	0.066 (2)	0.052 (2)	0.0387 (15)	0.0052 (17)	0.0024 (15)	−0.0013 (14)
C16	0.069 (2)	0.0491 (19)	0.0478 (16)	0.0015 (18)	0.0004 (16)	−0.0055 (15)
C17	0.062 (2)	0.0390 (16)	0.0510 (17)	0.0047 (15)	−0.0014 (15)	−0.0112 (14)
N1	0.099 (3)	0.0337 (16)	0.0359 (16)	0.0023 (17)	−0.0036 (15)	−0.0014 (13)
N2	0.0819 (18)	0.0439 (15)	0.0501 (14)	0.0088 (15)	−0.0110 (17)	−0.0058 (13)
N3	0.096 (2)	0.0393 (16)	0.0557 (16)	−0.0058 (15)	−0.0125 (15)	0.0036 (13)

### Geometric parameters (Å, °)

**Table:**

Br1—C1	1.905 (3)	C10—C11	1.415 (4)
C1—C6	1.360 (4)	C11—C13	1.400 (4)
C1—C2	1.363 (4)	C11—C12	1.428 (4)
C2—C3	1.381 (4)	C12—N3	1.139 (4)
C2—H2	0.9300	C13—C14	1.387 (4)
C3—C4	1.375 (4)	C13—C17	1.487 (4)
C3—H3	0.9300	C14—C15	1.509 (4)
C4—C5	1.393 (4)	C15—C16	1.521 (4)
C4—C7	1.484 (4)	C15—H15A	0.9700
C5—C6	1.386 (4)	C15—H15B	0.9700
C5—H5	0.9300	C16—C17	1.513 (4)
C6—H6	0.9300	C16—H16A	0.9700
C7—C14	1.376 (4)	C16—H16B	0.9700
C7—C8	1.432 (4)	C17—H17A	0.9700
C8—C10	1.408 (4)	C17—H17B	0.9700
C8—C9	1.426 (4)	N1—H1A	0.82 (3)
C9—N2	1.144 (3)	N1—H1B	0.85 (3)
C10—N1	1.346 (4)		
			
C6—C1—C2	122.2 (3)	C10—C11—C12	121.6 (2)
C6—C1—Br1	118.7 (2)	N3—C12—C11	177.4 (3)
C2—C1—Br1	119.1 (3)	C14—C13—C11	121.0 (3)
C1—C2—C3	118.8 (3)	C14—C13—C17	112.0 (2)
C1—C2—H2	120.6	C11—C13—C17	127.0 (3)
C3—C2—H2	120.6	C7—C14—C13	121.0 (2)
C4—C3—C2	121.2 (3)	C7—C14—C15	129.2 (3)
C4—C3—H3	119.4	C13—C14—C15	109.8 (2)
C2—C3—H3	119.4	C14—C15—C16	104.8 (2)
C3—C4—C5	118.3 (3)	C14—C15—H15A	110.8
C3—C4—C7	122.3 (2)	C16—C15—H15A	110.8
C5—C4—C7	119.4 (3)	C14—C15—H15B	110.8
C6—C5—C4	120.8 (3)	C16—C15—H15B	110.8
C6—C5—H5	119.6	H15A—C15—H15B	108.9
C4—C5—H5	119.6	C17—C16—C15	108.1 (2)
C1—C6—C5	118.6 (3)	C17—C16—H16A	110.1
C1—C6—H6	120.7	C15—C16—H16A	110.1
C5—C6—H6	120.7	C17—C16—H16B	110.1
C14—C7—C8	118.5 (2)	C15—C16—H16B	110.1
C14—C7—C4	121.4 (2)	H16A—C16—H16B	108.4
C8—C7—C4	120.1 (2)	C13—C17—C16	104.8 (2)
C10—C8—C9	119.6 (2)	C13—C17—H17A	110.8
C10—C8—C7	121.5 (2)	C16—C17—H17A	110.8
C9—C8—C7	118.9 (2)	C13—C17—H17B	110.8
N2—C9—C8	178.7 (3)	C16—C17—H17B	110.8
N1—C10—C8	121.3 (3)	H17A—C17—H17B	108.9
N1—C10—C11	121.0 (3)	C10—N1—H1A	121 (2)
C8—C10—C11	117.7 (2)	C10—N1—H1B	120 (2)
C13—C11—C10	120.1 (3)	H1A—N1—H1B	117 (3)
C13—C11—C12	118.3 (3)		
			
C6—C1—C2—C3	−0.4 (5)	N1—C10—C11—C13	178.8 (3)
Br1—C1—C2—C3	178.3 (2)	C8—C10—C11—C13	−2.5 (4)
C1—C2—C3—C4	0.2 (5)	N1—C10—C11—C12	−0.2 (5)
C2—C3—C4—C5	0.9 (4)	C8—C10—C11—C12	178.4 (3)
C2—C3—C4—C7	178.4 (3)	C13—C11—C12—N3	2(8)
C3—C4—C5—C6	−2.0 (4)	C10—C11—C12—N3	−179 (100)
C7—C4—C5—C6	−179.5 (3)	C10—C11—C13—C14	−0.5 (4)
C2—C1—C6—C5	−0.6 (5)	C12—C11—C13—C14	178.6 (3)
Br1—C1—C6—C5	−179.3 (2)	C10—C11—C13—C17	179.5 (3)
C4—C5—C6—C1	1.8 (4)	C12—C11—C13—C17	−1.4 (5)
C3—C4—C7—C14	−116.8 (3)	C8—C7—C14—C13	−0.8 (4)
C5—C4—C7—C14	60.6 (4)	C4—C7—C14—C13	178.0 (3)
C3—C4—C7—C8	62.0 (4)	C8—C7—C14—C15	−179.7 (3)
C5—C4—C7—C8	−120.6 (3)	C4—C7—C14—C15	−0.9 (5)
C14—C7—C8—C10	−2.4 (4)	C11—C13—C14—C7	2.3 (4)
C4—C7—C8—C10	178.8 (3)	C17—C13—C14—C7	−177.8 (3)
C14—C7—C8—C9	179.9 (3)	C11—C13—C14—C15	−178.7 (3)
C4—C7—C8—C9	1.1 (4)	C17—C13—C14—C15	1.3 (3)
C10—C8—C9—N2	28 (15)	C7—C14—C15—C16	173.9 (3)
C7—C8—C9—N2	−154 (15)	C13—C14—C15—C16	−5.0 (3)
C9—C8—C10—N1	0.3 (5)	C14—C15—C16—C17	6.8 (3)
C7—C8—C10—N1	−177.4 (3)	C14—C13—C17—C16	3.1 (3)
C9—C8—C10—C11	−178.3 (3)	C11—C13—C17—C16	−177.0 (3)
C7—C8—C10—C11	4.0 (4)	C15—C16—C17—C13	−6.1 (3)

### Hydrogen-bond geometry (Å, °)

**Table:**

*D*—H···*A*	*D*—H	H···*A*	*D*···*A*	*D*—H···*A*
N1—H1B···N2^i^	0.85 (3)	2.40 (3)	3.227 (4)	165 (3)
N1—H1A···N3^ii^	0.82 (3)	2.46 (3)	3.247 (4)	161 (2)

Symmetry codes: (i) −*x*+1, *y*+1/2, −*z*−1/2; (ii) −*x*+1, *y*−1/2, −*z*−1/2.
